# Optimization of Decolorization Process for Crude Polysaccharides from *Vicia villosa* Roth and Its Antioxidant and Growth-Promoting Activities

**DOI:** 10.3390/plants15132029

**Published:** 2026-06-30

**Authors:** Xiaoxiao Xiong, Xinyang Guo, Dongrui Zhang, Qiu Zhao, Ying Chang, Yan Li

**Affiliations:** 1 College of Life Sciences, Northeast Agricultural University, Harbin 150030, China; 19972771660@163.com (X.X.); s240902126@neau.edu.cn (X.G.); 2 College of Life Sciences, Heilongjiang University, Harbin 150080, China; dongruizhang96@hlju.edu.cn; 3 Tianjin Academy of Agricultural Sciences, Tianjin 300192, China; 4 Heilongjiang Academy of Black Soil Conservation and Utilization, Harbin 150086, China

**Keywords:** *Vicia villosa* Roth, polysaccharide, decolorization process optimization, antioxidant activity, plant growth-promoting activity

## Abstract

*Vicia villosa* Roth is an excellent green manure crop and offers promising applications in green agricultural production and bioactive substance exploitation. In this study, crude polysaccharides (VVP) were prepared from *V. villosa* using water extraction and alcohol precipitation. Single-factor experiments combined with Box–Behnken response surface methodology were employed to optimize the decolorization process of VVP with AB-8 macroporous resin. The optimal conditions were determined as follows: a resin-to-sample ratio of 1:140 (m/m), decolorization temperature of 78 °C, and a decolorization time of 300 min. Under these conditions, the decolorization rate reached 67.36% and the polysaccharide retention rate was 61.85%, with a relative error of only 2.1% between the model-predicted and experimental values. In vitro antioxidant assays showed that VVP possessed strong antioxidant activity. At a concentration of 2 mg/mL, the DPPH radical scavenging rate of VVP was 91.9%, and the ABTS+ radical scavenging rate reached 96% at 8 mg/mL. Moreover, VVP promoted crop growth and markedly alleviated the inhibition of salt stress on root development. To the best of our knowledge, this is the first report demonstrating that *V. villosa* crude polysaccharide extract simultaneously possesses strong antioxidant activity, salt-stress alleviation capacity, and plant growth-promoting effects, highlighting their potential as natural antioxidants and agricultural biostimulants.

## 1. Introduction

*Vicia villosa* Roth, an annual or biennial leguminous herb, is an excellent green manure and forage widely planted in temperate zones; after introduction into China, it has been popularized across Northeast, North and Northwest China. It can lower nitrogen loss, elevate soil organic carbon and ameliorate saline-alkali soil, forming an important agricultural biomass resource [1,2,3,4]. Current studies predominantly focus on its field agronomic application, whereas few studies concern the isolation and high-value exploitation of its endogenous bioactive constituents, restricting further resource development of this species.

Plant polysaccharides exert notable antioxidant and crop growth-promoting bioactivities. They upregulate SOD, CAT and other antioxidant enzymes to eliminate intracellular free radicals and mitigate oxidative injury [5]. Serving as natural biostimulants in agriculture, polysaccharides modulate endogenous hormone balance, improve photosynthesis and nutrient uptake, stabilize cell membranes, and activate stress-related pathways to strengthen plant tolerance against drought and salt stress [6,7,8,9]. Numerous polysaccharides from fungi, algae and medicinal herbs have been validated to boost crop growth and relieve abiotic damage, displaying promising prospects in green agriculture [10,11,12]. Leguminous green manures accumulate abundant polysaccharides, flavonoids and alkaloids, which are promising precursors for developing natural biostimulants [13,14]. Previous reports have proven the biofunctions of polysaccharide extracts from Sesbania, Melilotus and alfalfa on crop growth and salt resistance [15,16,17]. Plant polysaccharides possess the advantages of high safety and low toxicity, and are widely applied in food, pharmaceutical and ecological agriculture fields [18].

High-purity products are a prerequisite for polysaccharide functional research. Although water extraction-ethanol precipitation is the routine extraction approach, resultant crude polysaccharides contain massive pigment and protein impurities that interfere with purity detection and subsequent bioassay [19,20]. Hence, effective decolorization is critical for polysaccharide refinement. Accordingly, an efficient purification procedure is essential for the further development of polysaccharide resources. Previous laboratory studies found that crude *V. villosa* crude polysaccharide extract (VVP) contains substantial yellowish-brown pigments, which interfere with subsequent bioactivity analysis of VVP. Common strategies for polysaccharide decolorization include physical adsorption, oxidative degradation, organic solvent treatment and biological methods. Compared with other approaches, physical adsorption operates under mild conditions and maximizes the retention of polysaccharide bioactivity. As one of the most widely adopted techniques, macroporous adsorption resin chromatography exhibits prominent advantages, including mild reaction conditions, good selectivity and excellent reusability [21]. AB-8 weak-polar resin has an appropriate pore structure to adsorb pigments with low non-specific polysaccharide binding, and has achieved satisfactory decolorization on polysaccharides from *Rehmannia glutinosa* and seedless *Rosa roxburghii* [22,23], making it an ideal option for industrial decolorization of polysaccharides.

*V. villosa* is widely distributed with large biomass and represents a natural plant polysaccharide resource with great exploitation potential. In this study, crude VVP extracted by water extraction and alcohol precipitation was selected as the research material. For the first time, AB-8 macroporous resin was applied for decolorization and purification of VVP. Single-factor experiments combined with response surface methodology were used to optimize decolorization parameters, aiming to efficiently remove pigments while maintaining polysaccharide retention. On this basis, the in vitro DPPH and ABTS^+^ radical scavenging capacities of decolorized VVP were comprehensively evaluated, and its growth-promoting effect on crop seeds was also investigated. This study represents the first attempt to establish an efficient decolorization procedure for VVP and systematically clarify its dual antioxidant and plant growth-promoting properties, providing a theoretical basis and technical support for the further development and utilization of *V. villosa* resources. Meanwhile, the findings also enrich the research system on biological functions of polysaccharides derived from leguminous green manure crops.

## 2. Results

### 2.1. Analysis of Single-Factor Test Results

**Figure 1:**
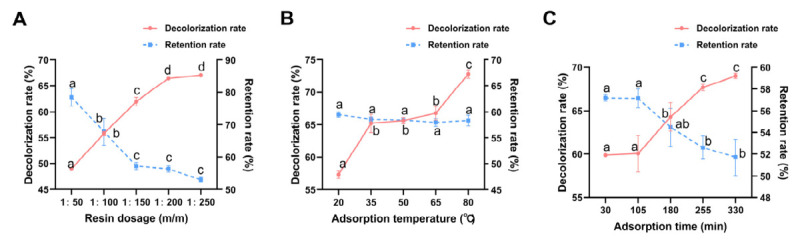
Single-factor experimental results of VVP polysaccharide decolorization. (**A**) resin dosage (m/m); (**B**) adsorption temperature (°C); (**C**) adsorption time (min). Data are presented as mean ± SD, *n* = 3. Different lowercase letters above error bars indicate statistically significant differences among groups at *p* < 0.05.

Figure 1A indicates that resin dosage markedly affects decolorization efficiency. Within the experimental range, the decolorization rate increased from 48.98% (1:50, m/m) to 66.49% (1:200, m/m) with increasing resin dosage, due to the enlarged contact area between polysaccharides and resin. Further dosage increase led to resin adsorption saturation and a slower rise in decolorization rate. Combined with the variation trend of polysaccharide retention rate, 1:150 (m/m) was selected as the central level for response surface design. Figure 1B shows that adsorption temperature greatly influenced the decolorization rate but had a slight effect on polysaccharide retention rate. Rising temperature reduced the viscosity of polysaccharide and pigment molecules and enhanced molecular activity, thus improving resin adsorption efficiency. The decolorization rate increased rapidly at 20–35 °C, remained stable at 35–65 °C, and rose again at 65–80 °C. Accordingly, 65 °C was set as the central temperature level. Figure 1C reveals that adsorption time obviously affected decolorization performance. Longer adsorption time prolonged contact between resin and pigment and improved decolorization rate. However, excessive incubation induced non-specific adsorption of polysaccharides, and thus decreased the retention rate. The rising trend of decolorization rate slowed obviously after 255 min, while the polysaccharide retention rate remained stable at 51.71–57.13% across the whole time range. Therefore, 255 min was chosen as the central time level.

### 2.2. Analysis of Box-Behnken Test Results

#### 2.2.1. Box-Behnken Experimental Design and Regression Model Analysis

Response surface methodology (BBD) was applied to clarify the interactive effects of various factors and optimize the decolorization parameters. The Box-Behnken design results are shown in Table S1. Based on single-factor experiments, resin dosage (A), decolorization temperature (B) and decolorization time (C) were selected as independent variables. A three-factor and three-level Box-Behnken design was conducted with the comprehensive score (Y) as the response value. The quadratic regression equation was established as follows: Y = 60.43 − 0.2325A + 0.8688B + 0.4338C − 2.06AB − 2.63AC + 0.2975BC − 3.27A^2^ + 0.1877B^2^ + 0.0828C^2^.

ANOVA results of the regression model are presented in Table 1. The regression model was extremely significant (*p* < 0.0001), and the lack of fit was insignificant (*p* > 0.05), indicating good model fitness and high experimental reliability. The determination coefficient R^2^ was 0.9751, adjusted R^2^ was 0.9430, predicted R^2^ was 0.9211, the precision was 17.1948, and the coefficient of variation (CV) was 1.01%. Decolorization temperature (B), quadratic term of resin dosage (A^2^), and interactive terms AB and AC showed significant effects on the comprehensive score (*p* < 0.05). The influence order of each factor was ranked as follows: resin dosage > decolorization temperature > decolorization time.

#### 2.2.2. Analysis of the Interaction of Various Factors via Response Surface Plots

**Figure 2:**
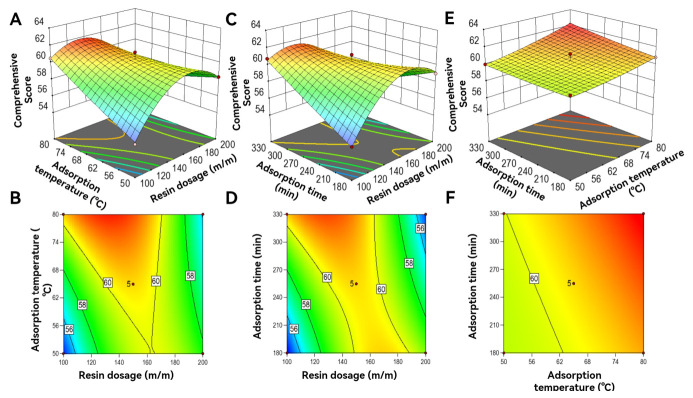
Response surface plots of the effects of variables on VVP decolorization efficiency. (**A**,**B**) resin dosage and adsorption temperature; (**C**,**D**) resin dosage and adsorption time; (**E**,**F**) adsorption temperature and adsorption time. The upper panels are 3D response surface plots, and the lower panels are corresponding contour plots.

The steepness of the response surface reflects the interaction intensity between factors: a steeper slope indicates a more significant impact on the comprehensive score. Contour plots indicate interaction significance: elliptical contours mean significant interactions, while circular contours mean insignificant ones. As shown in Figure 2A–D, the response surfaces of AB and AC are steep with elliptical contours, indicating significant interactions. Increasing temperature within 50–80 °C improved the decolorization effect, and the comprehensive score first increased and then decreased as resin dosage ranged from 1:100 to 1:200. As shown in Figure 2E,F, the BC response surface is gentle with nearly circular contours, indicating an insignificant interaction, consistent with ANOVA results.

#### 2.2.3. Verification of Prediction Model

Response surface analysis indicated that the optimal decolorization conditions for VVP were as follows: resin dosage of 1:138.78 (m/m), decolorization time of 305.35 min, and decolorization temperature of 77.8 °C. Under these conditions, the model-predicted comprehensive score was 63.3%. Considering practical operational feasibility, the parameters were adjusted to resin dosage of 1:140 (m/m), decolorization time of 300 min, and decolorization temperature of 78 °C. Three parallel validation experiments were carried out, and the results showed that the average decolorization rate of VVP was 67.36%, the polysaccharide retention rate was 61.85%, and the comprehensive score was 64.61%. The relative error between the actual value and the predicted value was only 2.1%, indicating high prediction accuracy of the model and reliable results. In addition, FT-IR spectra of VVP before and after decolorization were obtained and are presented in Supplementary Figure S3. Both spectra exhibited characteristic absorption bands commonly observed in plant polysaccharides, including a broad O–H stretching vibration around 3400 cm^−1^, C–H stretching vibration near 2920 cm^−1^, and strong carbohydrate-associated absorption bands in the 1000–1200 cm^−1^ region. These results provide preliminary evidence for the polysaccharide-rich nature of VVP and suggest that the major functional group characteristics were largely preserved after decolorization. Consistent with these FT-IR findings, the phenol–sulfuric acid assay showed that the polysaccharide purity increased from 58.8% in the crude extract to 73.4% after AB-8 decolorization, confirming a marked enrichment of the polysaccharide fraction.

### 2.3. DPPH and ABTS^+^ Radical Scavenging Capacity of VVP

**Figure 3:**
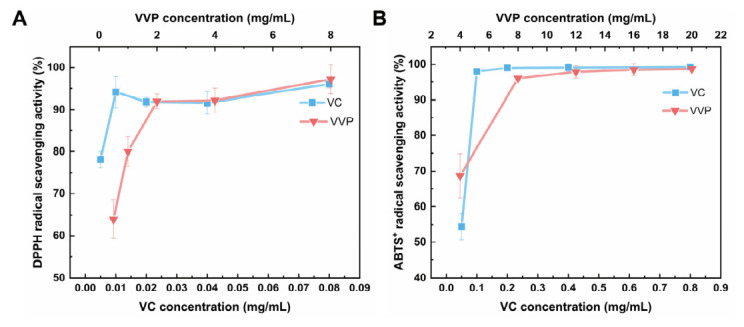
Radical scavenging abilities of VVP and VC. (**A**) DPPH radical scavenging ability; (**B**) ABTS^+^ radical scavenging ability. Data are presented as mean ± SD, *n* = 3.

As shown in Figure 3, the scavenging capacities of VVP for DPPH and ABTS^+^ radicals increased in a concentration-dependent manner. In Figure 3A, the DPPH radical scavenging rate of VVP rose rapidly with increasing concentration, reaching 91.9% at 2.0 mg/mL, which was comparable to the scavenging effect of 0.02 mg/mL VC. In Figure 3B, the ABTS^+^ radical scavenging rate of VVP increased with concentration, exceeding 90% at 8 mg/mL, indicating that VVP has excellent in vitro antioxidant activity.

### 2.4. Growth-Promoting Capacity of VVP

#### 2.4.1. Effects of VVP on Crop Growth Under Non-Salt Stress

**Figure 4:**
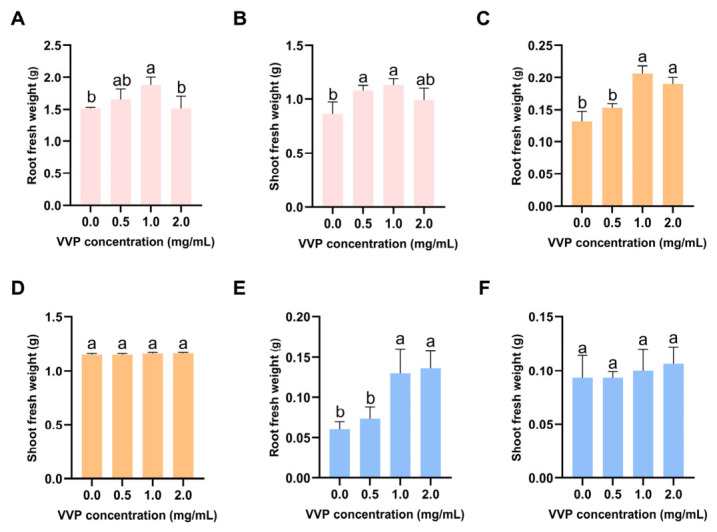
Effects of VVP on crop growth under non-salt conditions. (**A**,**B**) Root fresh weight and shoot fresh weight of maize; (**C**,**D**) Root fresh weight and shoot fresh weight of cucumber; (**E**,**F**) Root fresh weight and shoot fresh weight of rice. Data are presented as mean ± SD, *n* = 4. Different lowercase letters above error bars indicate statistically significant differences among groups at *p* < 0.05.

Under non-stress conditions, VVP exhibited an organ-specific growth-promoting effect on maize, cucumber and rice seedlings. As shown in Figure 4A–D, maize and cucumber presented a typical dose-effect relationship, with growth promotion at low concentrations and a weakened effect at high concentrations, and the optimal concentration was 1 mg/mL. For maize, the fresh root and shoot weights increased significantly with the elevation of VVP concentration within the range of 0.5–1 mg/mL. At 1 mg/mL, root and shoot fresh weights increased by 23.7% and 30.0% compared with the control, respectively, while the growth-promoting effect declined at 2 mg/mL. For cucumber, the root fresh weight increased by 56.9% at 1 mg/mL relative to the control, whereas no significant difference was observed in shoot fresh weight. As illustrated in Figure 4E,F, rice showed a positive response to VVP across the entire experimental concentration range. At 2 mg/mL, its root fresh weight increased by 139.0% compared with the control, which was higher than the increment of shoot fresh weight, and no high-concentration inhibition effect was detected.

#### 2.4.2. Effects of VVP on Crop Growth Under Salt Stress

**Figure 5:**
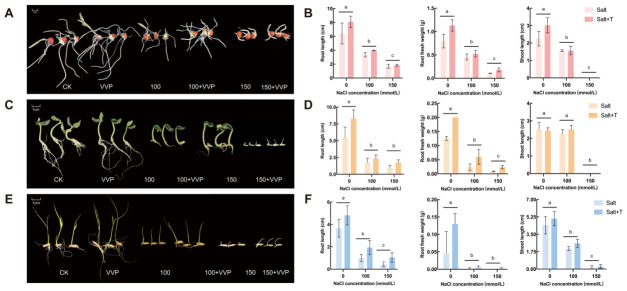
Effects of VVP on crop growth under salt stress. (**A**) Growth phenotype of maize; (**B**) Root length, total root fresh weight and shoot length of maize; (**C**) Growth phenotype of cucumber; (**D**) Root length, total root fresh weight and shoot length of cucumber; (**E**) Growth phenotype of rice; (**F**) Root length, total root fresh weight and shoot length of rice. Data are presented as mean ± SD, *n* = 4. Different lowercase letters above error bars indicate statistically significant differences among groups at *p* < 0.05.

Under salt stress, VVP effectively alleviated the growth inhibition of maize, cucumber and rice seedlings induced by NaCl, mainly by promoting root development, and the response patterns varied distinctly among different crops. As shown in Figure 5A,B, under 0–150 mmol/L NaCl stress, VVP increased the root length of maize by 9.0–26.1% and root fresh weight by 15.0–71.4%. The shoot length was significantly improved only under non-salt conditions, indicating that VVP enhanced the salt tolerance of maize primarily by promoting root growth. As shown in Figure 5C,D, for cucumber exposed to 0–150 mmol/L NaCl stress, VVP increased root length by 48.0–85.0% and root fresh weight by 60.0–159.0%. Shoot length was slightly promoted only at 100 mmol/L NaCl, suggesting that the growth-promoting effect was also mainly concentrated in the root system. As shown in Figure 5E,F, the root length of rice increased by 37.1–138.0% after VVP treatment, and the root fresh weight could recover to the normal level at 150 mmol/L NaCl. In conclusion, the salt tolerance regulation and growth-promoting effects of VVP on the three crops were mainly targeted at the root system.

## 3. Discussion

Leguminous green manures represent an important biomass resource in agricultural ecosystems. Polysaccharides, as abundant bioactive components in these plants, show promising potential for developing natural antioxidants and agricultural biostimulants. Based on the biological resources of *V. villosa*, this study applied AB-8 macroporous resin to decolorize its polysaccharide (VVP), achieving an optimal balance between decolorization efficiency and polysaccharide retention. Furthermore, the antioxidant and plant growth-promoting activities of VVP were verified for the first time, providing a theoretical basis for the exploitation and utilization of *V. villosa* polysaccharides.

### 3.1. Structural Context of Legume Polysaccharides and Relevance to VVP

To provide preliminary chemical characterization of the investigated material, FT-IR spectra of VVP before and after decolorization were obtained (Figure S3). Both spectra exhibited characteristic absorption bands commonly associated with plant polysaccharides, including broad O–H stretching vibrations, C–H stretching vibrations, and carbohydrate-related absorption bands. In particular, the prominent band centered in the 1600–1650 cm^−1^ region is diagnostically significant. This band is typically assigned to the asymmetric stretching vibration of COO^−^ in uronic acids, which is consistent with the fact that uronic acids are common constituents of legume polysaccharides as documented by Zhu et al. [14]. Given the measured purity of VVP (73.4%), a partial contribution from the amide I band of residual proteins cannot be excluded. Moreover, the major spectral features remained largely unchanged after decolorization, suggesting that the principal functional group characteristics of VVP were retained during the purification process. Legume polysaccharides have been widely reported as important bioactive constituents in Fabaceae plants. According to Zhu et al. [14], they are generally composed of glucose, galactose, arabinose, mannose, xylose, and uronic acids, although their composition varies among species and extraction procedures. Based on the FT-IR results and previous literature, VVP can be considered a polysaccharide-rich extract derived from *Vicia villosa*. The present study primarily focused on optimization of the decolorization process and biological activity evaluation, and further chemical characterization will contribute to a more comprehensive understanding of the composition and biological properties of VVP.

### 3.2. Decolorization Characteristics of VVP by AB-8 Macroporous Resin

As a weakly polar adsorbent, AB-8 macroporous resin has a suitable pore structure and surface hydrophobicity, which allows selective adsorption of pigment impurities while minimizing non-specific adsorption of polysaccharides. Therefore, AB-8 is widely recognized as an excellent medium for the refinement and purification of plant polysaccharides [24]. This study verified that resin dosage was the dominant factor affecting decolorization performance. Low resin dosage provides insufficient adsorption sites and leads to low decolorization efficiency, while excessive dosage causes co-adsorption of polysaccharides and consequently reduces polysaccharide retention. This trend is consistent with previous findings on polysaccharide decolorization from *Lycopus lucidus* Turcz. and *Andrographis paniculata* [25,26,27]. RSM revealed significant interactions among dosage, temperature, and time. After optimization, the decolorization rate reached 67.36% with a polysaccharide retention rate of 61.85%. The moderate retention can be attributed to unavoidable physical adsorption of polysaccharides onto the resin, co-adsorption inherent to static systems, and possible entrapment or co-precipitation. Comparable retention rates (55–75%) have been reported for plant polysaccharides decolorized by macroporous resins under similar conditions. Static decolorization has inherent efficiency limitations; future dynamic column chromatography could enhance both decolorization and polysaccharide recovery.

### 3.3. Antioxidant Activity of VVP and Its Association with Salt Stress Regulation

The crude VVP extract exhibited strong radical scavenging activity. Its DPPH scavenging rate reached 91.9% at 2 mg/mL, and ABTS^+^ scavenging activity exceeded 96% at 8 mg/mL, indicating that the crude extract possesses considerable antioxidant potential. Comparative analysis showed that the DPPH scavenging capacity of VVP was comparable to polysaccharides from coffee cherry peel and *Siraitia grosvenorii*, and markedly higher than those from *Asteris Radix* et Rhizoma and *Citrus reticulata* Blanco [28,29,30]. These two assays, which reflect the capacity to quench both lipophilic and hydrophilic radicals, are widely accepted as standard and complementary methods for evaluating the direct radical-scavenging ability of natural antioxidants. Polysaccharides are abundant in polar functional groups such as hydroxyl and carboxyl groups, which can serve as hydrogen and electron donors. These active groups directly eliminate free radicals and reactive oxygen species, and interrupt the chain reaction of lipid peroxidation. In addition, polysaccharides can chelate transition metal ions to restrain excessive ROS accumulation caused by the Fenton reaction, thereby alleviating oxidative stress through multiple regulatory pathways [31,32]. Although our current work mainly characterizes the direct in vitro radical-scavenging activity of VVP, exploring its regulatory roles on intracellular antioxidant enzymes including SOD, CAT and POD, under salt stress would help to elaborate its underlying functional mechanism more comprehensively. Relevant enzymatic experiments will be prioritized and systematically implemented in our subsequent research. Considering the physiological responses of plants to salt-induced oxidative stress, the excellent in vitro antioxidant performance of VVP lays a physiological foundation for relieving salt damage. It is inferred that VVP regulates plant salt tolerance by modulating antioxidant metabolism, thus alleviating salt stress injury and maintaining normal seedling growth.

### 3.4. Salt Tolerance and Growth-Promoting Mechanism as Well as Dose Effect of VVP

The growth-promoting effect of VVP on crop seedlings followed a typical dose-dependent pattern: low-concentration promotion and high-concentration inhibition, with 1 mg/mL determined as the optimal concentration. This trend is consistent with the action characteristics of other biostimulants such as chitosan and polysaccharides from *Ulva* [33,34]. Moreover, VVP displayed distinct organ-specific growth-promoting effects. Under both non-stress and salt-stress conditions, VVP exerted a much stronger promoting effect on roots than on shoots, which is highly consistent with the physiological feature that roots act as the primary responsive organ to salt stress [35,36]. The bioactive components within crude VVP (including polysaccharides and residual minor metabolites) possibly improve crop salt resistance via three pathways. First, it maintains cell membrane integrity and ion homeostasis via antioxidant regulation, thereby alleviating the toxic effects of salt ions. Second, it promotes root elongation and biomass accumulation, expands the absorption area of water and nutrients, and relieves salinity-induced physiological drought. Third, it regulates the synthesis of osmotic adjustment substances, improves cellular water retention, and enhances plant osmotic adaptation [6,37,38]. In this study, the growth-promoting efficiency of VVP declined at high concentrations. It is hypothesized that the high osmotic pressure of concentrated VVP solution may restrict root water uptake and disrupt nutrient absorption balance [39].

In conclusion, this study optimized the decolorization parameters of *V. villosa* polysaccharide (VVP) via response surface methodology, and confirmed that the crude VVP extract shows favorable antioxidant and crop growth-regulating activities. The results provide a technical and theoretical basis for subsequent structural characterization, functional evaluation, and comprehensive utilization of VVP. Nevertheless, the present study still has several limitations. First, individual components of VVP were not separated, purified, and structurally identified. Second, field validation and in-depth molecular mechanisms underlying its growth-promoting effects remain unclarified. Third, dynamic decolorization conditions were not optimized. Future research will adopt HPLC, FT-IR and NMR to characterize the chemical structure of VVP, optimize dynamic decolorization processes, and conduct field trials combined with physiological and biochemical measurements. These efforts will further elucidate the intrinsic mechanism of VVP in regulating plant growth and salt tolerance.

## 4. Materials and Methods

### 4.1. Materials and Reagents

Whole plants of *V. villosa* were collected from the MingZhu Experimental Zone (Harbin, China). Plant materials were air-dried, ground, and sieved through a 200-mesh sieve. Corn cultivar Yufeng 303, cucumber cultivar Cuizhenbao, and rice cultivar Wuyou Rice No. 4 were all purchased from corresponding professional seed companies. Methanol, n-butanol, potassium persulfate, papain (enzyme activity: 100,000 U/g), vitamin C (VC), 1,1-diphenyl-2-picrylhydrazyl (DPPH), 2,2′-azino-bis (3-ethylbenzothiazoline-6-sulfonate) (ABTS), dialysis bag (3500 Da) were used in this experiment. All reagents were of analytical grade and purchased from regular commercial suppliers.

### 4.2. Preparation of VVP

*V. villosa* samples were air-dried and ground. The samples were defatted with methanol three times at a solid–liquid ratio of 1:3 (g/mL), and the residues were air-dried. Distilled water was added to the residues at a solid–liquid ratio of 1:10 (g/mL), and the mixture was extracted in a water bath at 90 °C three times for 1 h each. All extracts were combined and concentrated. Absolute ethanol was added at a volume ratio of 1:3 (*v*/*v*), and the mixture was kept at 4 °C for 24 h to complete alcohol precipitation. The obtained precipitate was redissolved with distilled water and concentrated via rotary evaporation, followed by the addition of 0.8% (*w*/*v*) papain and enzymatic hydrolysis at 55 °C for 6 h. The Sevag method [40] (chloroform:n-butanol, 4:1, *v*/*v*) was used for deproteinization until no obvious white flocculent precipitate was observed after centrifugation. The polysaccharide solution was dialyzed against running water using a 3500 Da dialysis bag for 3 d, then lyophilized in a vacuum freeze dryer (YTLG-10A) to obtain crude polysaccharide extract (VVP) before decolorization. The VVP was prepared into a 2 mg/mL aqueous solution for subsequent decolorization optimization.

### 4.3. Optimization of Decolorization Process

#### 4.3.1. Single Factor Test

Three variables were selected for static adsorption tests [41,42]. The mass ratios of polysaccharide to macroporous resin (m/m) were set as 1:50, 1:100, 1:150, 1:200 and 1:250. Adsorption temperatures were 20 °C, 35 °C, 50 °C, 65 °C and 80 °C, and adsorption times were 30, 105, 180, 255 and 330 min. Each treatment was performed in three replicates.

The formulas are shown below. To determine the decolorization rate, the untreated VVP solution was scanned from 200 to 800 nm with a full-wavelength microplate reader (Model A51119700DPC). As shown in Figure S1, the maximum absorption peak was observed at 220 nm. Because this wavelength reflects the presence of various colored and conjugated impurities in the crude extract, absorbance at 220 nm was selected as an empirical indicator for evaluating impurity removal during the decolorization process. When absorbance values exceeded the reliable detection range of the spectrophotometer, samples were appropriately diluted prior to measurement. The reported absorbance values were subsequently corrected using the corresponding dilution factors. Accordingly, absorbance at 220 nm was determined to evaluate the overall removal of total conjugated impurities, and the decolorization rate was calculated via the following formula:

$$\mathrm{Decolorization}\;\mathrm{rate}\;=\;\frac{A_{1}\;-\;A_{2}}{A_{1}}\;\times \;100\%$$

In the formula, A_1_ and A_2_ are the absorbance of polysaccharide solution at 220 nm before and after decolorization, respectively.

Polysaccharide content was determined by the phenol-sulfuric acid method at 490 nm using a full-wavelength microplate reader (A51119700DPC). The glucose standard curve is shown in Figure S2, with a standard regression equation of Y = 5.0817X − 0.0063 and a correlation coefficient of R^2^ = 0.9935. The polysaccharide retention rate was calculated according to the following formula:

$$\mathrm{Retention}\;\mathrm{rate}\;=\;\frac{C_{4}}{C_{3}}\;\times \;100\%$$

In this formula, C_3_ and C_4_ represent polysaccharide mass concentrations before and after decolorization, respectively.

For the comprehensive weighted scoring method, referring to Ren et al. [23], both the decolorization rate and polysaccharide retention rate were assigned a weight of 0.5. The comprehensive score was calculated using the following formula:

Comprehensive score = (0.5 × X + 0.5 × Y) × 100

In the formula, X represents the decolorization rate; Y represents the polysaccharide retention rate.

#### 4.3.2. Box–Behnken Test

Based on single-factor results, Box–Behnken design (BBD) was used to optimize decolorization conditions. Macroporous resin dosage (A), temperature (B) and treatment time (C) were chosen as independent variables, with the comprehensive score as the response value. Each factor was set at three levels (−1, 0, 1), as shown in Table 2.

### 4.4. Evaluation of Antioxidant Activity

#### 4.4.1. Determination of DPPH Radical Scavenging Rate

The experiment was modified from Tian et al. [12]. Decolorized VVP was prepared into a series of concentrations (0.5, 1.0, 2.0, 4.0, and 8.0 mg/mL). 1 mL of the sample solution was mixed with 1 mL of 0.1 mmol/L DPPH-ethanol solution and incubated in the dark for 30 min. Absorbance (A) was measured at 517 nm. Absolute ethanol served as the blank control (A_0_, instead of sample solution) and sample blank (A_1_, instead of DPPH-ethanol solution). Vitamin C (VC) at concentrations of 0.005, 0.01, 0.02, 0.04, and 0.08 mg/mL was used as the positive control. Concentration gradients of VVP and VC were optimized via pre-experiments to reach the respective plateau stage of antioxidant reaction. Each treatment was in triplicate, and the scavenging rate was calculated using the following formula:

$$\mathrm{DPPH}\;\mathrm{radical}\;\mathrm{scavenging}\;\mathrm{rate}\;=\;\frac{A_{0}\;-\;(A\;-\;A_{1})}{A_{0}}\;\times \;100\%$$

#### 4.4.2. Determination of ABTS^+^ Radical Scavenging Rate

The assay was modified from Roberta et al. [43]. ABTS^+^ working solution was prepared by mixing equal volumes of 7 mmol/L ABTS^+^ solution and 2.45 mmol/L potassium persulfate solution, and incubated in the dark for 12 h. The mixture was diluted with PBS buffer to an absorbance of approximately 0.7 at 734 nm. Decolorized VVP was prepared into a series of concentrations (4, 8, 12, 16, and 20 mg/mL). 1 mL of the sample solution was mixed with 1 mL of ABTS^+^ working solution and incubated in the dark for 15 min. Absorbance (A) was measured at 734 nm. PBS buffer served as the blank control (A_0_, replacing sample solution) and sample blank (A_1_, replacing ABTS^+^ working solution). VC at concentrations of 0.05, 0.1, 0.2, 0.4, and 0.8 mg/mL was used as the positive control. Concentration gradients of VVP and VC were optimized via pre-experiments to reach the respective plateau stage of antioxidant reaction. Each treatment was run in triplicate, and the ABTS^+^ radical scavenging rate was calculated using the following formula:

$${\mathrm{ABTS}}^{+}\;\mathrm{radical}\;\mathrm{scavenging}\;\mathrm{rate}\;=\;\frac{A_{0}\;-\;(A\;-\;A_{1})}{A_{0}}\;\times \;100\%$$

### 4.5. Evaluation of Growth-Promoting Activity

#### 4.5.1. Growth-Promoting Test of VVP Under Non-Salt Stress

Corn, cucumber and rice seeds were disinfected in 1% sodium hypochlorite for 10 min, rinsed thoroughly and soaked at room temperature for 8 h. Fifteen seeds were placed on double-layer gauze per Petri dish, and 5 mL of VVP solutions at different concentrations were added. The experiment was set with four groups (*n* = 4): blank control (distilled water), 0.5 mg/mL, 1 mg/mL and 2 mg/mL VVP treatment groups. All groups were incubated at 25 °C under a 18/6 h light/dark cycle, with daily water supplementation to keep moisture stable. 8 seedlings were randomly selected from each group for the determination of total fresh root and bud weight.

#### 4.5.2. Growth-Promoting Test Under Salt Stress

Corn, cucumber and rice seeds were disinfected in 1% sodium hypochlorite for 10 min, rinsed thoroughly and soaked at room temperature for 8 h. Fifteen seeds were placed on double-layer gauze per Petri dish, and 5 mL of treatment solutions with different concentrations of NaCl and VVP were added. The experiment was set with six groups (*n* = 4): blank control (distilled water), 1 mg/mL VVP, 100 mmol/L NaCl, 100 mmol/L NaCl + 1 mg/mL VVP, 150 mmol/L NaCl, and 150 mmol/L NaCl + 1 mg/mL VVP. All groups were incubated at 25 °C under a 18/6 h light/dark cycle, with daily water supplementation to keep moisture stable. Eight seedlings were randomly selected from each group to determine total fresh root weight, root length and bud length, and representative seedlings were photographed for phenotype.

### 4.6. Data Analysis

Data processing and graphing were performed using Excel 2021 and GraphPad Prism 10.1.2. One-way analysis of variance (ANOVA) with Tukey’s multiple comparison was applied, and differences at *p* < 0.05 were considered statistically significant. Design-Expert 13.0 was used for response surface analysis.

## 5. Conclusions

This study investigated the biological activities of a crude polysaccharide-rich extract obtained from *Vicia villosa.* After decolorization and refinement with AB-8 macroporous resin, VVP displayed multiple biological activities: it efficiently scavenged DPPH and ABTS^+^ free radicals in vitro and exerted favorable physiological regulation on tested crops. At appropriate concentrations, VVP markedly promoted seedling growth of maize, cucumber and rice and alleviated salt-induced root growth inhibition, resulting in obvious increases in root length and root fresh weight within the tested concentration range. For the first time, this study confirms that VVP possesses diversified bioactive characteristics including antioxidant capacity, plant growth promotion and salt stress mitigation, which is consistent with the original research hypothesis. These findings provide essential theoretical support for high-value utilization of *Vicia villosa* resources and the development of eco-friendly agricultural biostimulants, contributing to the sustainable development of green agriculture. Further systematic research will focus on structural characterization of VVP and its field application performance.

## Figures and Tables

**Table 1:** Results of Analysis of Variance in the Box-Behnken Trial. * Indicates significant difference at *p* < 0.05 level.

Source	Sum of Squares	Degree of Freedom	Equal Square	F	*p*
Model	98.08	9	10.90	30.42	<0.0001 *
Resin dosage (A)	0.4324	1	0.4324	1.21	0.3083
Adsorption temperature (B)	6.04	1	6.04	16.85	0.0045 *
Adsorption time (C)	1.51	1	1.51	4.20	0.0796
AB	16.89	1	16.89	47.15	0.0002 *
AC	27.67	1	27.67	77.22	<0.0001 *
BC	0.3540	1	0.3540	0.9881	0.3533
A^2^	45.15	1	45.15	126.03	<0.0001 *
B^2^	0.1484	1	0.1484	0.4143	0.5403
C^2^	0.0288	1	0.0288	0.0805	0.7849
Residual	2.51	7	0.3583		
Undrafted item	0.2781	3	0.0927	0.1663	0.9138
Pure error	2.23	4	0.5575		
Total	100.59	16			
Model sufficiency parameters	Comprehensive score				
R^2^	0.9751				
Adj-R^2^	0.9430				
Pre-R^2^	0.9211				
Precision	17.1948				
Coefficient of variation	1.01				

**Table 2:** Factors and Levels of BBD-Behnken Test.

Level	Factor		
	A (Resin Dosage, Polysaccharide: Resin, m/m)	B (Adsorption Temperature/°C)	C (Adsorption Time/min)
−1	1:100	50	180
0	1:150	65	255
1	1:200	80	330

## Data Availability

Data are contained within the article.

## Supplementary Materials

The following supporting information can be downloaded at: https://www.mdpi.com/article/10.3390/plants15132029/s1, Figure S1: Full-wavelength scanning spectrum of crude VVP solution before decolorization in the range of 200–800 nm. Samples exhibiting absorbance values beyond the reliable detection range of the spectrophotometer were diluted prior to measurement, and the absorbance values shown in the spectrum were corrected using the corresponding dilution factors; Figure S2: Glucose standard curve; Figure S3: FTIR spectra before and after decolorization; Table S1: Box-Behnken Design and Results.

## Abbreviations

The following abbreviations are used in this manuscript:

VVP	*Vicia villosa* Roth polysaccharides
