# Supplementary material for: Optimization of Decolorization Process for Crude Polysaccharides from Vicia villosa Roth and Its Antioxidant and Growth-Promoting Activities
Source: Plants (Basel). 2026 Jun 30;15(13):2029. doi: 10.3390/plants15132029 (PMC13363838; doi:10.3390/plants15132029)
Supplement: Supplementary file 1 [file plants-15-02029-s001.zip › plants-4359467-supplementary.pdf]

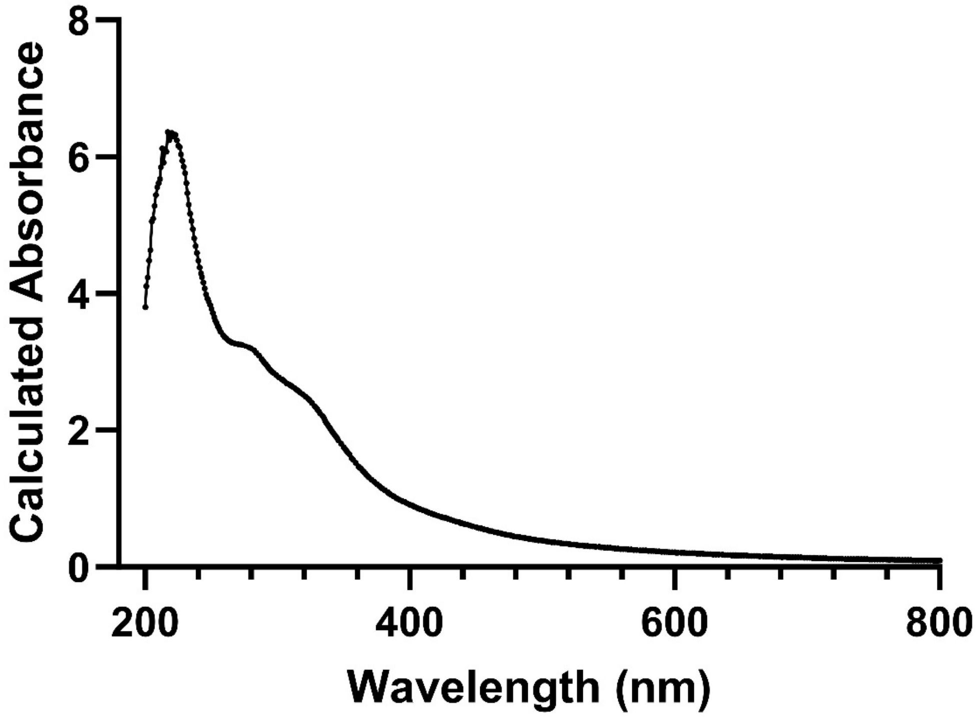

Fig. S1. Full-wavelength scanning spectrum of crude VVP solution before decolorization in the range of 200–800 nm. Samples exhibiting absorbance values beyond the reliable detection range of the spectrophotometer were diluted prior to measurement, and the absorbance values shown in the spectrum were corrected using the corresponding dilution factors.

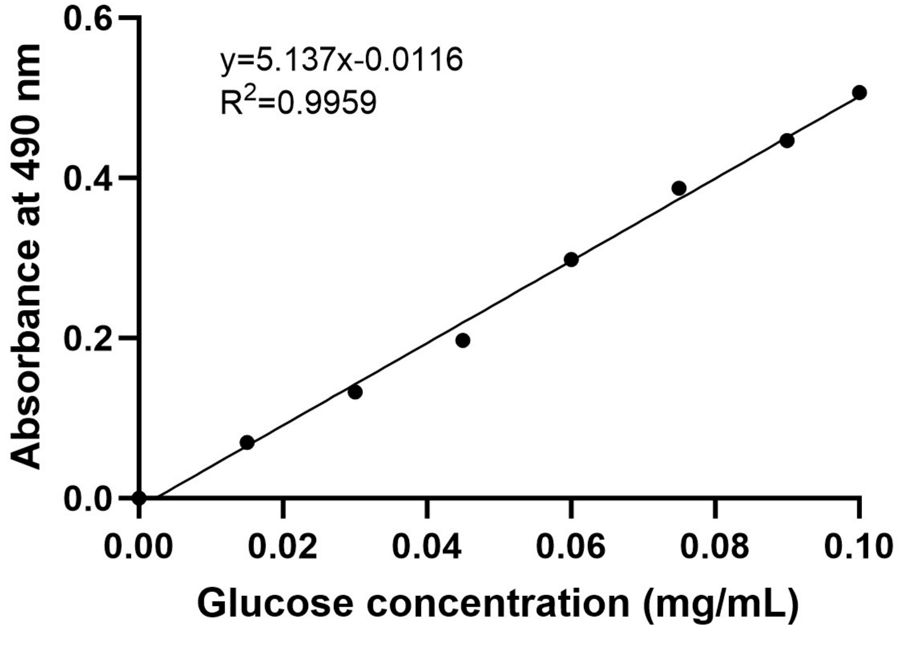

Fig. S2. Glucose standard curve.

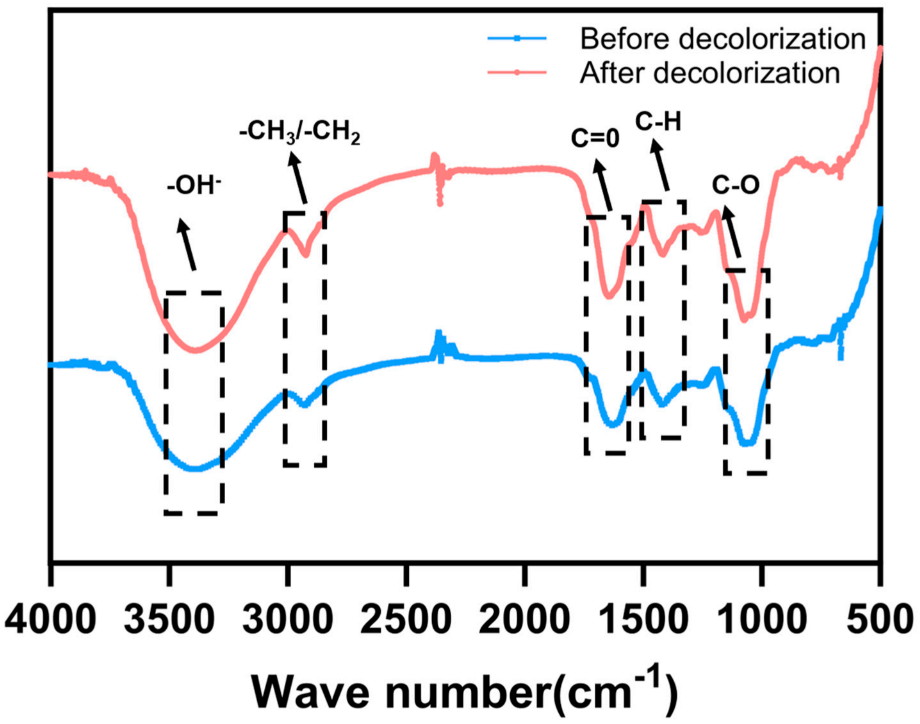

Fig. S3. FTIR spectra before and after decolorization.

Table S1. Box-Behnken Design and Results.

Group	Actual variable range			Comprehensive score	
	Resin dosage(m/m)	Adsorption temperature(°C)	Adsorption time(min)	Actual value	Predicted value
1	100	50	255	54.42	54.61
2	200	50	255	58.3	58.26
3	100	80	255	60.5	60.46
4	200	80	255	56.16	55.89
5	100	65	180	54.44	54.47
6	200	65	180	59	59.26
7	100	65	330	60.74	60.60
8	200	65	330	54.78	54.88
9	150	50	180	59.9	59.26
10	150	80	180	60.81	60.99
11	150	50	330	60	60.13
12	150	80	330	62.1	61.87
13	150	65	255	58.92	60.56
14	150	65	255	61.2	60.56
15	150	65	255	59.17	60.56
16	150	65	255	60.26	60.56
17	150	65	255	61.21	60.56
